# Chondrocytes supplemented to bone graft-containing scaffolds expedite cranial defect repair

**DOI:** 10.1038/s41598-023-46604-z

**Published:** 2023-11-06

**Authors:** Idan Carmon, Anna Zobrab, Michael Alterman, Rami Tabib, Adir Cohen, Leonid Kandel, Alexander Greenberg, Eli Reich, Nardi Casap, Mona Dvir-Ginzberg

**Affiliations:** 1 Laboratory of Cartilage Biology, Institute of Bio-Medical and Oral Research, Faculty of Dental Medicine, Hebrew University of Jerusalem, Jerusalem, Israel; 2 Department of Maxillofacial Surgery, Faculty of Dental Medicine, Hadassah-Hebrew University, P. O. Box 12272, 9112102 Jerusalem, Israel; 3 Joint Replacement and Reconstruction Unit, Orthopedic Surgery Complex, Hadassah-Hebrew University Medical Center at Mount Scopus, Jerusalem, Israel

**Keywords:** Regeneration, Transdifferentiation, Cell biology, Pathogenesis

## Abstract

Critical maxillofacial bone fractures do not heal spontaneously, thus, often there is a need to facilitate repair via surgical intervention. Gold standard approaches, include the use of autologous bone graft, or devices supplemented with osteogenic growth factors and bone substitutes. This research aimed to employ a critical size calvaria defect model, to determine if the addition of chondrocytes to collagen-containing bone graft substitute, may expedite bone repair. As such, using a critical size rat calvaria defect, we implanted a collagen scaffold containing bone graft substitute (i.e., Bone graft scaffold, BG) or BG supplemented with costal chondrocytes (cBG). The rats were subjected to live CT imaging at 1, 6, 9, and 12 weeks following the surgical procedure and sacrificed for microCT imaging of the defect site. Moreover, serum markers and histological evaluation were assessed to determine osseous tissue regeneration and turnover. Live CT and microCT indicated cBG implants displayed expedited bone repair vs, BG alone, already at 6 weeks post defect induction. cBG also displayed a shorter distance between the defect edges and greater mineral apposition distance compared to BG. Summerizing, the data support the addition of chondrocytes to bone substitute, accelerates the formation of new bone within a critical size defect.

## Introduction

Maxillofacial critical bone defects are highly prevalent, especially after trauma^1^, and constitute a serious clinical burden. Reconstructive surgical interventions often consist of an autologous bone graft, which is surgically integrated into the defect site^2, 3^. However, this procedure entails risk of donor site morbidity following autologous bone grafting. To overcome this obstacle, other approaches have been employed to regenerate bone comprising of tailored-devices based on titanium, filled with bone regenerative materials, as collagen-based bone substitutes and growth factors^4^. Although bone is regenerated effectively using this approach, titanium is non-degradable and may cause mucosal damage^5^, possibly leading to further future complications.

In order to effectively generate new bone, a composite transplant must provide osteoconductive properties to timely replace the graft with new lamellar bone, while simultaneously harboring osteogenesis promoting capacities. Demineralized freeze-dried bone allograft (DFBA) is often utilized in fracture repair, to enable mechanical durability at the affected site, while bone is replaced^6^. These materials either contain or are combined with other osteoinducive growth factors as Bone Morphogenic Protein 2 (BMP2), as well as others^7^. Notably, reports support the use of such DFBA constructs as osteoinducive in clinical and preclinical models^8–10^. This study attempts to assess if the addition of chondrocytes to a DFBA containing collagen constructs, may expedite bone regeneration in a rat critical size defect.

Recent reports support the use of chondrocytes, which are derived from an aneural and avascular tissue, to generate a bone block^11–13^. While cartilage -as a cell source- is less explored, its aneural and avascular nature may less predispose to donor site morbidity than autologous bone grafting^14^. Resident cartilage cells- chondrocytes- are easy to isolate, propagate and maintain their capacity to ossify at early passage^13^. Chondrocytes are inherently capable of ossifying during physiological and pathological conditions (i.e., osteoarthritis, chondrocalcinosis and fracture repair) as well as during skeletal development^15, 16^. Hence, use of chondrocytes for maxillofacial repair is an exciting and a feasible approach. This study utilized Bone Graft substitute (denoted BG), which is composed of a clinically approved collagen matrix paired with demineralized freeze dried bone particles (i.e., DFBA), and compared to the same BG implant seeded with chondrocytes (denoted cBG). Unlike other studies, we adopted the use of a clinically approved collagen scaffold, given that it is expected to effectively and timely remodel with bone formation and resorption, potentially leading to gradual formation of lamellar bone in the defect site. The significance of this study lies on the understanding that DFBA grafts may constitute of residual growth factors^17^, which may prevent chondrocytes from mineralizing, as we previously reported^13^. Collectively, the data show that chondrocytes added to DFBA are capable of expediting bone repair, as previously shown for such a model employing collagen scaffolds containing chondrocytes alone^13^.

## Materials and methods

### Rapid human chondrocyte isolation

All procedures were performed with Hadassah Medical Center Institutional Review Board approval and in accordance with the Helsinki Declaration ethical principles for medical research involving human subjects (0488-09-HMO). Following obtaining an informed consent from end-stage osteoarthritis (OA) donors, articular cartilage was obtained from the knee joints of OA patients undergoing total knee arthroplasty (total n = 6, mean age: 73.3 years, mean body mass index 31.09 kg/m^2^).

**Figure 1:**
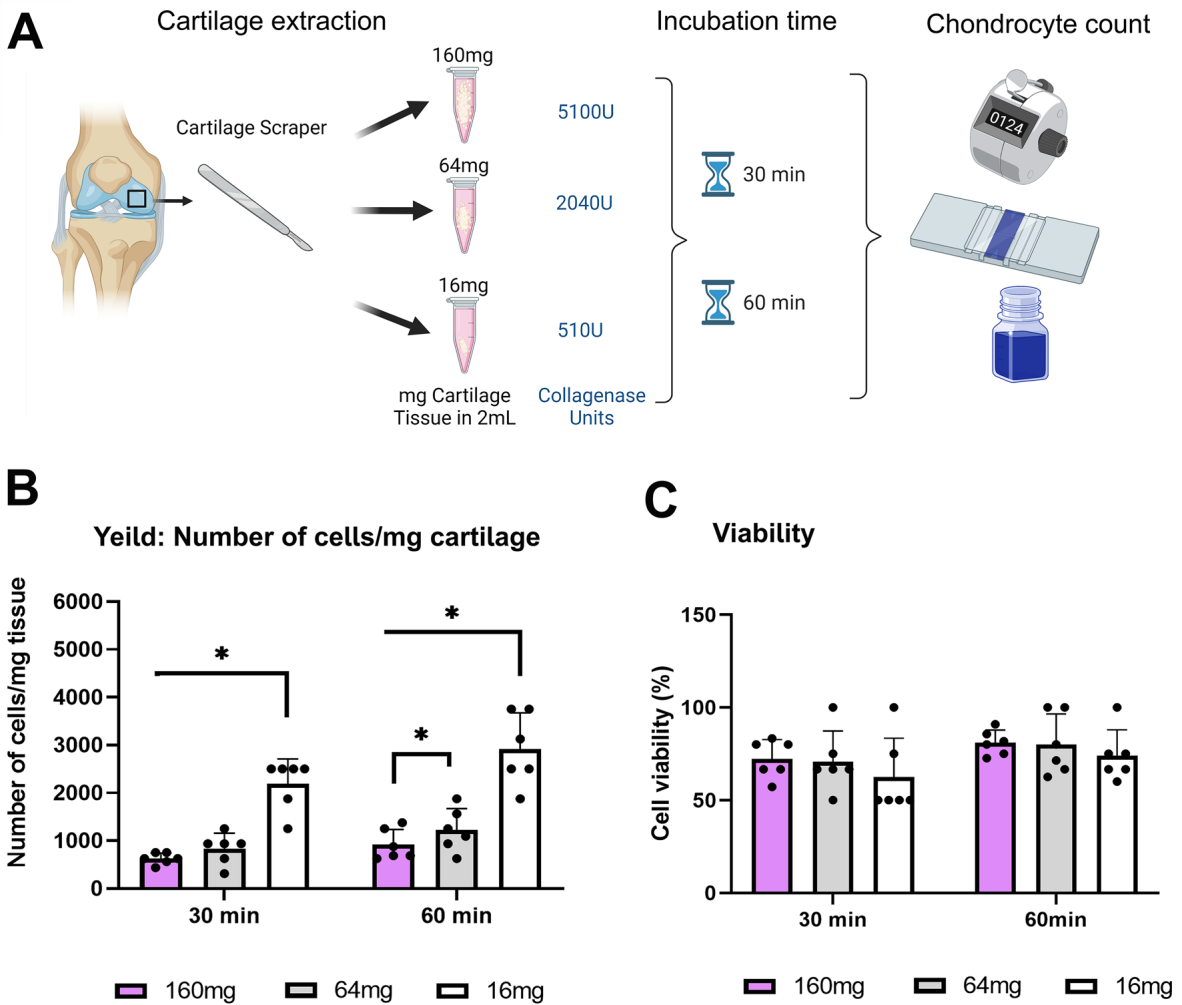
Rapid isolation of human chondrocytes. (**A**) Illustration of rapid human chondrocytes isolation protocol. Using clinically in-use bone scraper, we weighted 160, 64 and 16 mg of OA-derived human articular cartilage and incubated with collagenase 31.875 units/mg tissue for 30 or 60 min. Isolated cells were counted for (**B**) cell yield per mg tissue and (**C**) percent of cell viability using Trypan blue exclusion assay. Statistical significance was assessed based on Wilcoxon test comparison (*p* < 0.05 denoted “*”, n = 6).

To assess if chondrocytes can be isolated in a potential surgical setting, for immediate application and without the need to culture the isolated cells, we characterized a cell isolation protocol. Accordingly, scraped articular cartilage from intact sites, were obtained using a clinically in-use SafeScraper^®^ TWIST (i.e., bone scraper, Geistlich Pharma #40600). The sample was weighed to 160, 64 and 16 mg, incubated with collagenase II (Worthington, #LS004177) at 31.875 units/mg tissue for 30 or 60 min to isolate cells (Scheme in Fig. 1A). Next, cells were counted using Trypan blue (T8154, Merck-Sigma Aldrich, Rahway, NJ), exclusion assay to calculate cell yield and viability.

### Isolation of rat costal chondrocytes and generation of chondrocyte-seeded scaffolds

**Figure 2:**
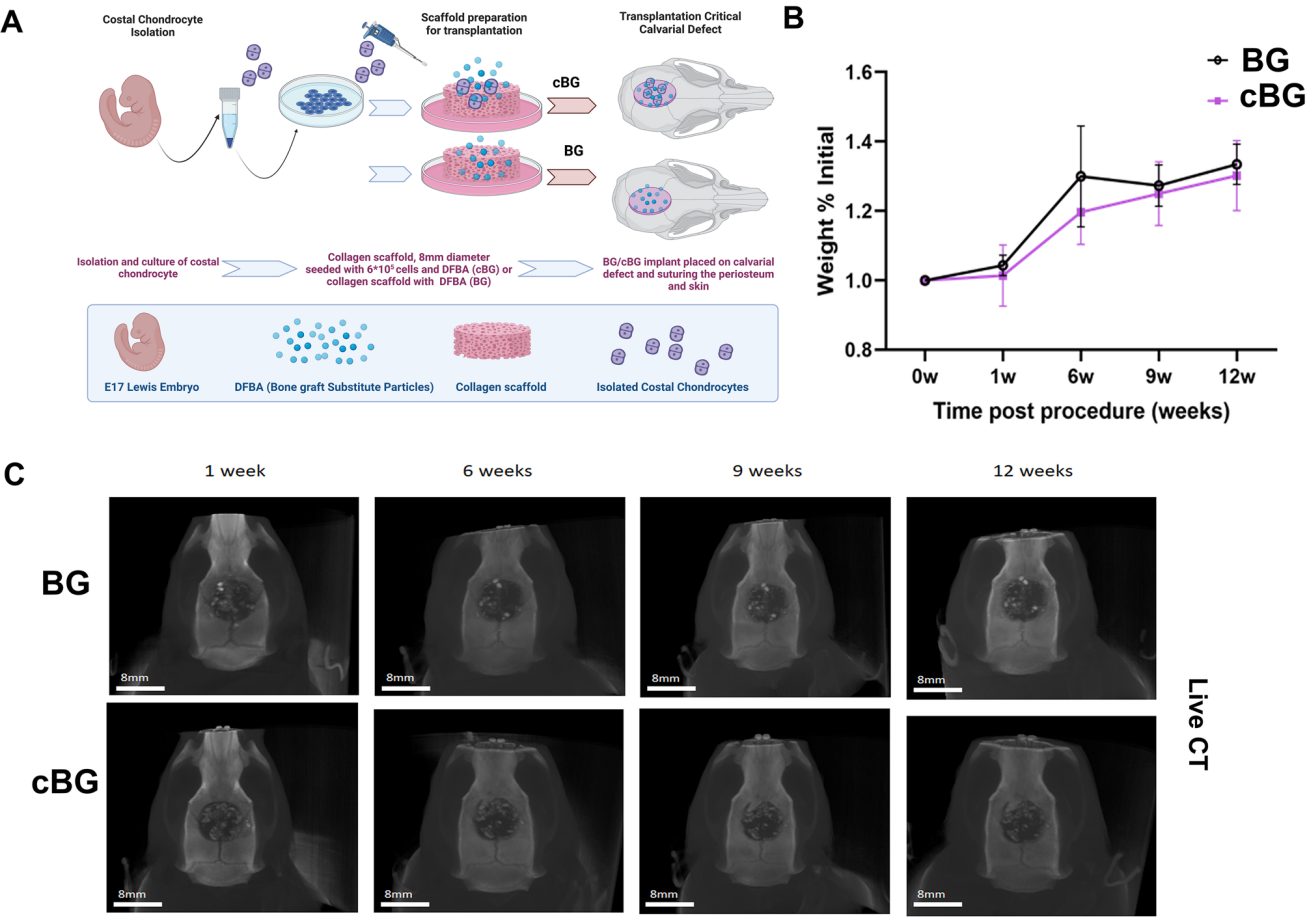
Assessing the effect of chondrocyte seeded collagen scaffold in a critical size defect calvaria model. (**A**) Illustration of the experimental model employed. E17 costal chondrocytes were isolated and propagated for 48 h and seeded into Zimmer Collagen Tape scaffolds of 0.9 * 0.9 cm^2^ in enriched growth media, as indicated in Materials and Methods. As control, collagen scaffolds devoid of cells were soaked in enriched growth media and transplanted onto the defect. Prior to transplantation 1.3 mg of DFBA bone graft (OraGraft; LifeNet Health) was added on top of the implanted scaffold. The scaffolds were placed as the cell-seeded surface facing upwards, towards the skin following suturing of the connective tissue and skin over the implant. During the experimental duration the rats were weighed (**B**) and subjected to Live CT imaging (**C**) at 1 week baseline, 6, 9 and 12-weeks post-surgery. BG denotes the implants comprized of bone graft (DFBA)-containing collagen scaffold, while cBG denotes the implanted containing chondrocyte-seeded collagen scaffolds with bone graft. Statistical significance was assessed based on Wilcoxon test (*p* < 0.05 denoted “*”, n = 7). Panel A was adapted from Carmon et al. 2023^13^.

Costal chondrocytes from rat E17 embryos were isolated, and processed as illustrated in Fig. 2A. As part of the procedure the pregnant rats was anesthetized using (0.02 mg/kg Ketamine and 100 mg/kg Xylazine; Merck-Sigma Aldrich, Rahway, NJ) and sacrificed at Day 17 of pregnancy. The embryos were isolated, and ribcage was extracted based on protocol by Gosset et al.^18^. Next, thoracic cage incubated with 0.2% collagenase (Worthington, #LS004177), 37 °C, 2 h followed by additional overnight incubation with 0.05% collagenase. Isolated cells were filtered and cultured in DMEM (Merck-Sigma Aldrich, Rahway, NJ) growth media supplemented with 1% Insulin-transferrin-selenium (ITS; IBMH, Beit Haemek, Israel), 50 µg/mL Ascorbic acid (Merck-Sigma Aldrich, Rahway, NJ) and 0.1 mM/L β-glycerophosphate (i.e., “enriched DMEM growth media” or “eDMEM”), as previously described^13^. 48 h prior to seeding the cells on top of the collagen scaffolds (Zimmer© collagen Tape, 0.9 * 0.9 cm^2^, Zimmer Biomet, IN), eDMEM was replaced with enriched BioMPM (i.e., eBioMPM, IBMH, Beit Haemek, Israel), containing the same supplements.

### Seeding costal chondrocytes on collagen scaffolds

Collagen scaffolds (Zimmer© collagen Tape, 0.9 * 0.9 cm^2^ each) were submersed for 3 h in eBioMPM growth media (approx. 10 mL) in 37 °C. Then, 20 µL of 1 * 10^6^ chondrocytes in eBioMPM were seeded on top of the soaked scaffold and set for 1 h, 37 °C, to let cells slowly penetrate into the scaffold. Next, 150 µL of eBioMPM was added to each well for 24 h (total of 170 µL per well, 1 * 10^6^ cells, 0.9 cm^2^ scaffold). On the following day, the scaffolds were transplanted on top of a rat calvarial critical size defect and supplemented with 1.3 mg cortical bone graft (DFBA, OraGraft; LifeNet Health, VA; #DGC1/4), due to the physical restriction enabling the containment of the cells and graft within the scaffold milieu. The scaffold seeding surface faced the periosteum of the critical size defect. As control, we used empty collagen scaffolds supplemented with bone graft denoted BG, while costal chondrocyte-containing scaffold with bone graft denoted cBG (see illustration in Fig. 2A). The use of bone graft particles without a collagen scaffold, posed risk of dissemination, which is why this study did not use this as a control. Moreover, given that our interested lied in understanding the dynamic between the bone graft and chondrocytes, we did not use a collagen scaffold or an untreated control herein.

### Rat critical size defect model

Experimental procedures involving Lewis rats were carried out in accordance with NIH Committees for animal use and care (ARAC guidelines) and based on AAALAC (Association for Assessment and Accreditation of Laboratory Animal Care International) and in adherence with the ARRIVE guidelines. Hebrew University Institutional Animal Care and Use Committee approved the study protocol (MD-20-16095-4). Rats (male, n = 7) were grown in 12-h light/dark cycles and received food and water ad libitum. Following randomization, an eight mm diameter critical size calvarial defect was employed in 3-month Lewis rats (males) as described by Spicer et al.^19^, with slight modifications. As mentioned, following trauma, the rats were implanted with BG, or cBG (Fig. 2A illustration). Pain control medication was provided three days after surgery (I.P Buprenorphine 0.01–0.05 mg/kg; Merck-Sigma Aldrich, Rahway, NJ). Moreover, to monitor bone formation we administered subcutaneous (SC) Alizarin-Complexon (25 mg/kg; #A3882; Merck-Sigma Aldrich, Rahway, NJ) at 2 weeks post-surgery and Calcein (25 mg/kg, #C0875; Merck-Sigma Aldrich, Rahway, NJ) was administered at 6-weeks post procedure^19^.

#### Live computed tomography (CT) scan and analysis

The rats were monitored for weight and activity 3 times a week for the 12-week duration and sacrificed at 12 weeks post defect induction. In vivo live Three dimensonal (3D) CT scans of the rat skull were acquired at 1, 6, 9 and 12 weeks following skull defect procedure, as previously reported in Carmon et al.^13^. All scans were acquired using a Siemens Inveon preclinical small-animal PET/CT scanner (Siemens Healthcare^®^, Knoxville, TN, USA) on isoflurane anesthetized rats. All images were acquired under the following settings: 95 μm pixel size, 360 projections, 300 ms exposure, 80 Kvp, with a volume of interest (VOI) of 494.88 mm^3^. 3D reconstructions of the skull of the rats were evaluated using Inveon Research Workplace software (Siemens, Germany), to determine mineral content in a set area encompassing the defect. Bone volume (mm^3^) was extracted and calculated in net values per time, or cumulative values per indicated scan, wherein the previous measures were summed to a single bone volume value for the time of measurement (e.g.cumulative bone volume of week 9 is composed of the sum for weeks 1, 6 and 9).

#### Post-sacrifice microcomputed tomography (µCT)

Following sacrifice, calvarias were fixed in 4% paraformaldehyde solution (PFA, Merck-Sigma Aldrich, Rahway, NJ) for 72 h and subsequently stored in 70% EtOH for microCT (µCT) assessment of calcified tissue (µCT 40 Scanco-medical, 70 kV, 115uA, 10 µm pixel size; Scanco, Switzerland), as previously reported in Carmon et al.^13^. Scans were reconstructed using Dragonfly software (ORS, Montreal, Canada) and the area of interest was selected (i.e., defected area; 8 mm diameter 3D cylindrical shaped). 3D analysis was assessed to quantify Bone Volume (mm^3^) and Bone volume fraction (i.e., Bone Volume/Total Volume; %BV/TV) using the Dragonfly software tool Wizard Bone Analysis. Notably, the CT and µCT scans were unable to differentiate between the bone graft particles and regenerated osseous matrix. As such, each data point represents existing bone graft, as well as mineralized matrix generated within the defect.

#### Serum analysis of bone turnover markers

Rat sera was collected at endpoint, diluted 1:10 and sera levels of Cross Linked C-telopeptide of type I collagen (CTX-1, cat# E-EL-R1456, Elabscience, TX), Procollagen type I N-Terminal Propeptide (PINP, cat# MBS2506450, MyBioSource, SD), were measured via ELISA assay kit, in accordance to manufactures protocol.

#### 3Histological analysis of calvaria

Rat calvaria were collected and fixed in 4% PFA for 72 h, then transferred to 70% ethanol for short term storage. Samples were submersed in 4% PFA, when shipped AnaPath Services GmbH (Liestal, Switzerland), for histological processing and histopathological evaluation, as previously reported in Carmon et al.^13^. Fixation, storage, and transport were conducted in a light shielded environment. The implantation sites were processed by methyl methacrylate (MMA, Merck-Sigma Aldrich, Rahway, NJ) resin embedding, sawed coronally at the center of the defect, using a diamond band saw (EXAKT System, Poland). Samples were next ground and polished to a final thickness of approximately 40–60 µm (EXAKT System, Poland) according to AnaPath Services GmbH SOP’s.

The sections were stained for Paragon staining (toluidine blue and basic fuchsine, AnaPath proprietary staining) or captured unstained for fluorescence imaging (Olympus VS200 Slide scanner, with a VS-304 M camera; 20 × magnification; Olympus, Japan). Paragon staining detects ranges of mineralized tissue in shades of pink depending on degree of mineralization. Particularly, non-mineralized osteoid stains blue, however mature bone stains light pink while lesser degree of matrix mineralization appears in a darker shade of pink.

Histopathology scoring was carried out by a blinded pathologist according to a semi-quantitative histopathology evaluation scoring method based on the adapted ISO 10993-6 scoring (Supplemental Tables 1, 2). Briefly, using the ISO 10993-6:2016(E) method; a score difference between 0.0 and 2.9 is considered no or minimal host reaction, 3.0 to 8.9 slight host reaction, 9.0 to 15.0 moderate host reaction and ≥ 15.1 severe host reaction compared to a reference material. Scoring was adapted to reflect less central sectioning of the defect wherein the defect distance is reduced. Images were captured with an Olympus BX 46 camera, and defect distance measured as illustrated in Fig. 5f. Raw scoring analysis is presented in Supplemental Table 3. Notably, five random calvaria defect samples were chosen from the seven biological samples for histological analysis.

### Analysis of bone formation

Mineral Apposition Distance (MAD) was determined following SC administration of Alizarin-complexon (red fluorescence staining) and 25 mg/kg Calcein‏ (green fluorescence staining) at 2 and 6 weeks, respectively, post-operative stages. The distance between the red and green line were measured in three separate areas of the slide and averaged on a graph for analysis (µm distance)—(i) native bone (i.e., intact calvaria bone, at least 3 mm distance from defect edge); (ii) defect edge (i.e., the interface between the intact bone and the defect); and (iii) defect zone (Bone graft islands within the defect site), as seen in Fig. 4C illustration. Each zone was captured at × 20 followed by × 4 magnification and triplicates of measurements were carried out per area (Fig. 4C; zones i–iii). Triplicate zonal measurements (i.e., zones i, ii, iii) were averaged and analyzed. Within the defect site, the cumulative net area of the bone graft islands were calculated (µm^2^) and cumulative surface of bone graft was determined (mm), manually. Additionally, we performed manual tracing of the accumulated surface of fluorescent stained deposited osteoid, surrounding the bone graft particles. Finally, the distance between the defect edges, as seen in Paragon stained histological sections was measured using the average length between the edges after employing three, equally-spaced, parallel lines, or using area contouring of the defect. The data were acquired using image J software (NIH) and Olympus OlyVIA software (Olympus, Japan). Notably, five random calvaria defect samples were chosen from the seven biological samples for MAD analysis.

#### Statistical analysis

Each experiment was repeated at least three times (n $\ge$ 3) and the average and standard deviation calculated per group. In-vitro rodent analysis was carried out for live CT, microCT and serum analysis for n = 7, while histology and fluorescent labeling was carried out for n = 5 subset of the cohort. All the data were analysed via non-parametric One-way ANOVA, followed by Wilcoxon test for paired groups, assuming statistical significance of *p* < 0.05. Pearson correlation was carried out assuming a confidence level greater than 95% (*p* < 0.05), to be significant. Notably, Pearson’s correlation (R) that is closer to 1 indicates a good fit to linear regression, while values closer to 0 indicate weak fit to linear regression. Regression (R^2^) indicates the variation around the linear regression line. All schemes were generated via BioRender Software, and graphs and statistical assays via GraphPad Prism 9.0.

## Results

### Simulation of chondrocyte yields from expedited cell isolation procedure

As a first step, we aimed to assess the potential applicability of using a critical mass of primary chondrocytes in a surgical setting, wherein they are to be rapidly harvested from donor site and introduced in an autologous host site. As such, we attempted to determine the yields and viability of scraped OA-derived articular chondrocytes following collagenase incubation of 30 or 60 min*. *In a previous report^20^, nasal bovine cartilage was isolated using an equivalent of 30 units collagenase per mg cartilage with 600 cell yield per mg, following 1 h agitation*.*

Here we isolated 16, 64 and 160 mg of articular cartilage tissue with a bone scraper and subjected these samples to collagenase (31.8 units/mg tissue, i.e., total 510, 2035 and 5100 units, respectively), as illustrated in Fig. 1A. The results show that at both time-frames, reduced tissue weight per reaction, increased cell yield per mg tissue with some preference towards the 60 min incubation (Fig. 1B). Furthermore, we did not detect significant variations in the resulting cell viability, in all samples and incubation procedures, (Fig. 1C).

### Addition of chondrocytes to bone graft-containing scaffolds displayed increased bone volume

As a first step we carried out critical size calvarial bone defect (8 mm diameter), which was transplanted with bone graft, seeded within collagen scaffold (BG), or chondrocyte-seeded scaffold supplemented with bone graft (cBG) (Fig. 2A). Monitoring the rats during the 12-week experimental duration did not exhibit changes in weight gain between groups (Fig. 2B).

**Figure 3:**
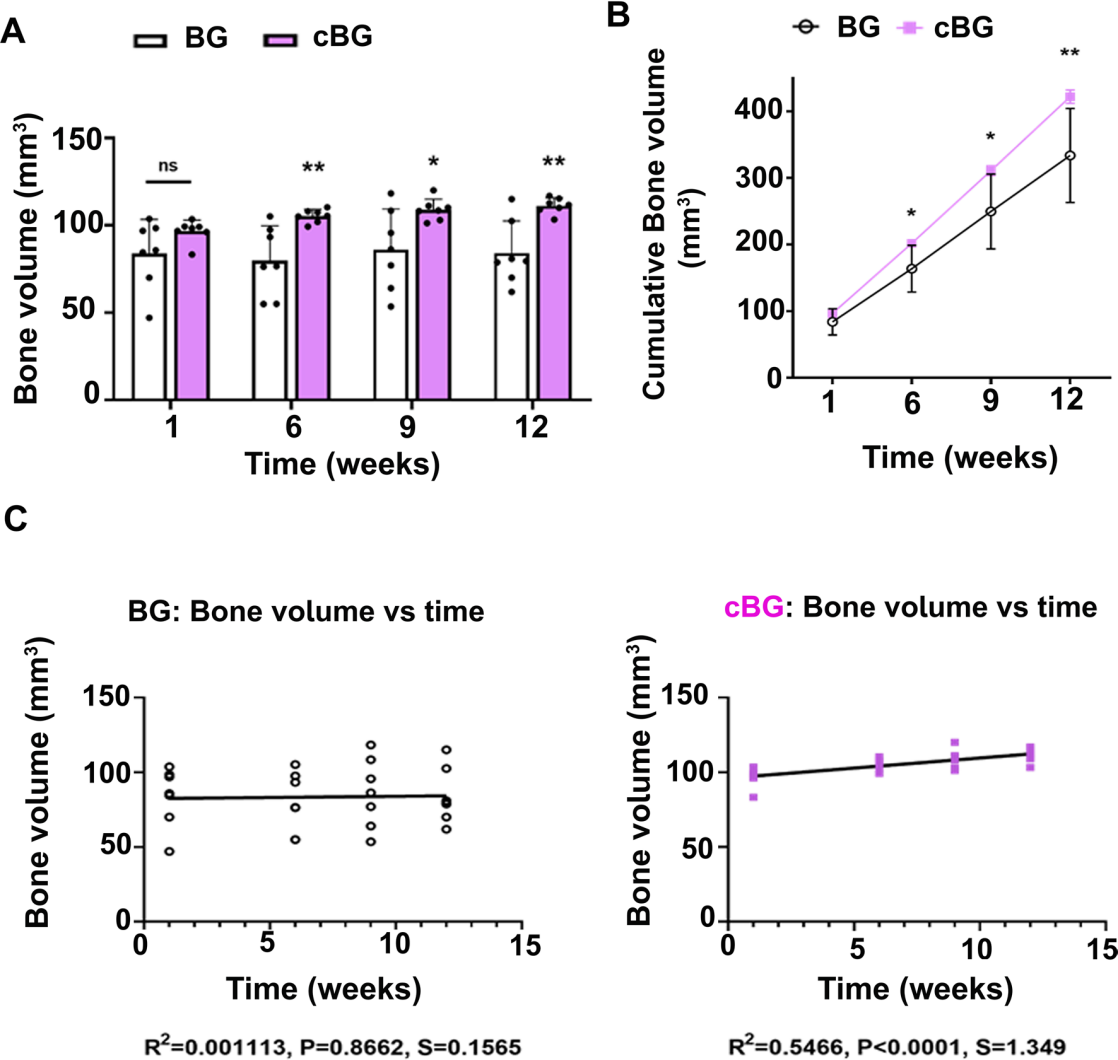
Bone quantification at the defect site was enhanced when bone graft was supplemented with chondrocytes. (**A**) Depicts quantitative Live CT bone volume (mm^3^). (**B**) depicts cumulative bone volumes ( mm^3^) from 1 week baseline to 12 weeks endpoint of Live CT scans for BC and cBC. One way ANOVA was carried for BC and cBC, each with *p* < 0.0001. (**C**) Depicts a correlation between time in weeks post procedure versus bone volume (mm^3^) for BG (left-handed panel) and cBG (right-handed panel). One way ANOVA was carried for BC (*p* = 0.93336), and cBC (*p* = 0.0009). Each graph depicts R^2^, P-value and a slope (S) based on Pearson correlation. Statistical significance between the BG and cBG was assessed based on Wilcoxon test (panels A, B) or Pearson correlation (panel C) (*p* < 0.05 denoted “*”, *p* < 0.01 denotes “**”, n = 7).

Live CT carried out during the experimental duration, exhibited that both treatments displayed time dependent increase in bone volume within the defect (Fig. 2C, representative images). However, closer analysis of live CT defect site, exhibited increased bone volume (Fig. 3A) in cBG group already 6 weeks post defect transplantation, which was also apparent in cumulative values per time point (Fig. 3B).

**Figure 4:**
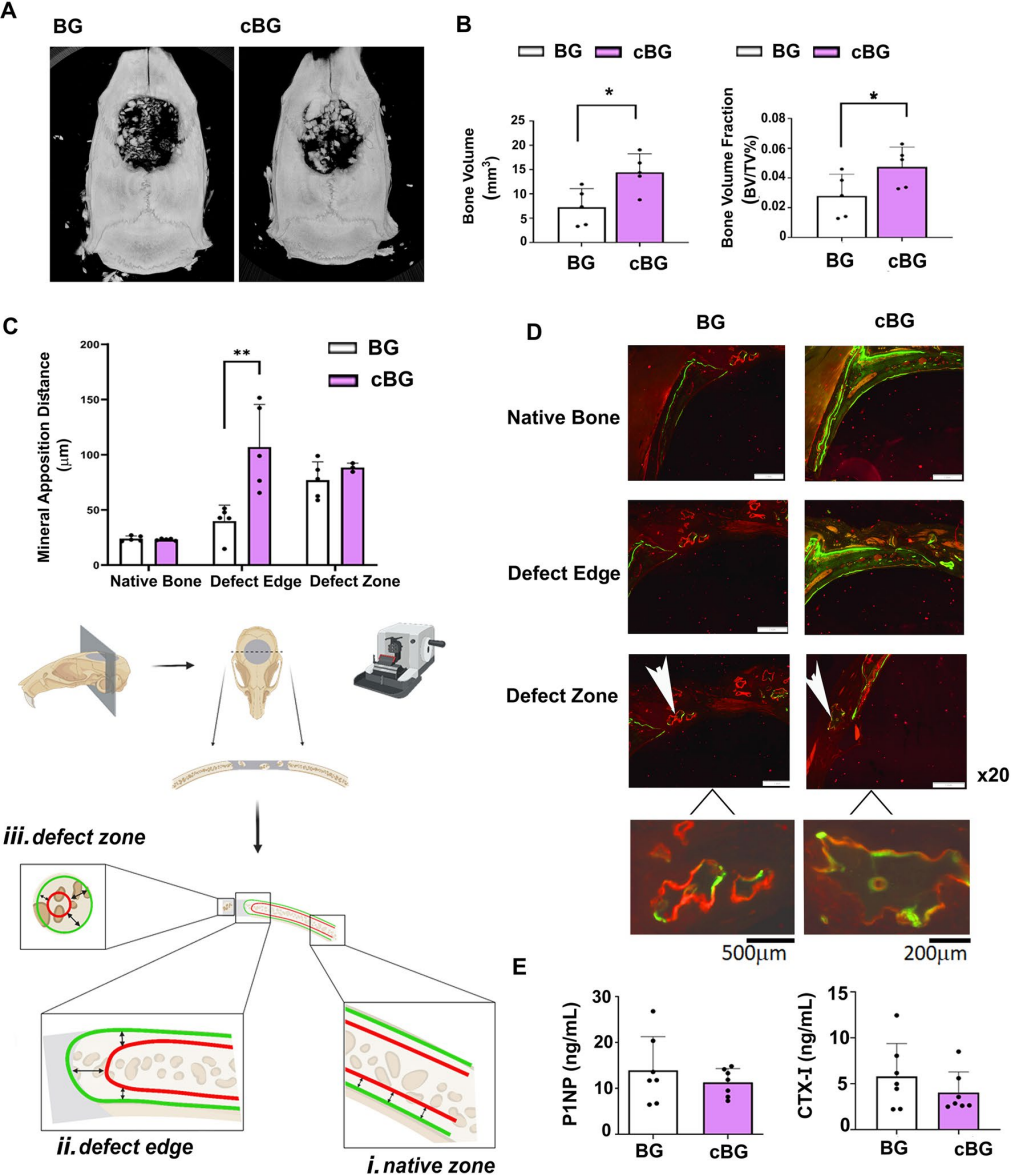
MicroCT and immunofluorescent (IF) analysis confirm an anabolic response which is unsupported in serum bone markers analysis. Rats were subjected to the experimental procedure described in 2A, sacrificed 12 weeks post-procedure and subjected to microCT (n = 7). (**A**) Depicts representative microCT images. (**B**) Depicts microCT analysis of bone volume (mm^3^), left-handed panel, and bone volume fraction (BV/TV%), right-handed panel, of BG and cBG. (**C**) Depicts mineral apposition distance (MAD) following Alizarin-complexon and Calcein injections at 2- and 6-weeks post procedure (n = 5). MAD was calculated at 3 areas: (i)–(iii), as specified in Materials and Methods. (**D**) Representative IF images of the three denoted areas (× 20). The arrow point at an area of the osseous tissue within the defect site (possibly bone allograft), which is enlarged in the image underneath. At 12 weeks endpoint, serum was collected and analyzed for (**E**) bone anabolic marker, Procollagen type I N-Terminal Propeptide (P1NP), left-handed panel, and bone catabolic marker, Cross Linked C-telopeptide of type I collagen (CTX-1), right-handed panel, to assess bone turnover. Statistical significance was assessed based on Wilcoxon test (*p* < 0.05 denotes as “*”). Panel C illustration was adapted from Carmon et al. 2023^13^.

Correlating between time and bone volume calculated in Live CT scans exhibited a steeper slope and a significantly stronger correlation coefficient in the cBG group compared to the correlations of the BG transplanted group (Fig. 3C), indicating that addition of chondrocytes expedited bone formation. In line with this data, microCT analysis of 12-week endpoints confirmed higher bone volume in the cBG implanted group vs BG implanted group (Fig. 4A, B). Notably, the microCT bone readout was one order of magnitude lower than the live CT, given the variation in the scanning settings and the ROI, which included more native bone for the live CT due to the use of a square-shaped setting around the defect.

### cBG constructs exhibit increased bone formation at defect edges

Additional analysis of bone formation was done by assessing the distance between Alizarin-complexon (red fluorescence staining) and Calcein (green fluorescence staining), which are florescent compounds that bind newly formed bone. The compounds were administered 4 weeks apart (2- and 6-weeks post procedure) and denote the distance between newly formed bone within a 4 weeks’ timeframe. In particular, the longer the distance, the faster the bone is formed. The mineral apposition distance (MAD) between green and red was measured in mid-coronal sections of the defect, for three regions, as listed in the scheme in Fig. 4C (below the plot). One region is the native bone at the right side of the defect (Fig. 4C; denoted “i”) the defect edge (Fig. 4C; denoted “ii”) and the mid area of the defect zone (Fig. 4C; denoted “iii”). For each zone we measured 3 consistent sites as shown in the scheme (Fig. 4C, lower panel, black arrows), which were averaged. The data show that only the defect edge region exhibited a significant increase in MAD for the cBG vs the BG graft (Fig. 4C; representative images in Fig. 4D).

Analysis of serum bone markers for anabolism, Procollagen type I N-Terminal Propertied (P1NP) shows the level of bone formation was unchanged (Left graph, Fig. 4E). Similarly, levels of bone catabolic marker in serum catabolic (i.e., Cross Linked C-telopeptide of type I collagen; CTX-1) also remained unchanged between the treatments (Right graph, Fig. 4E).

### Histological analysis of defect for host response and bone formation

**Figure 5:**
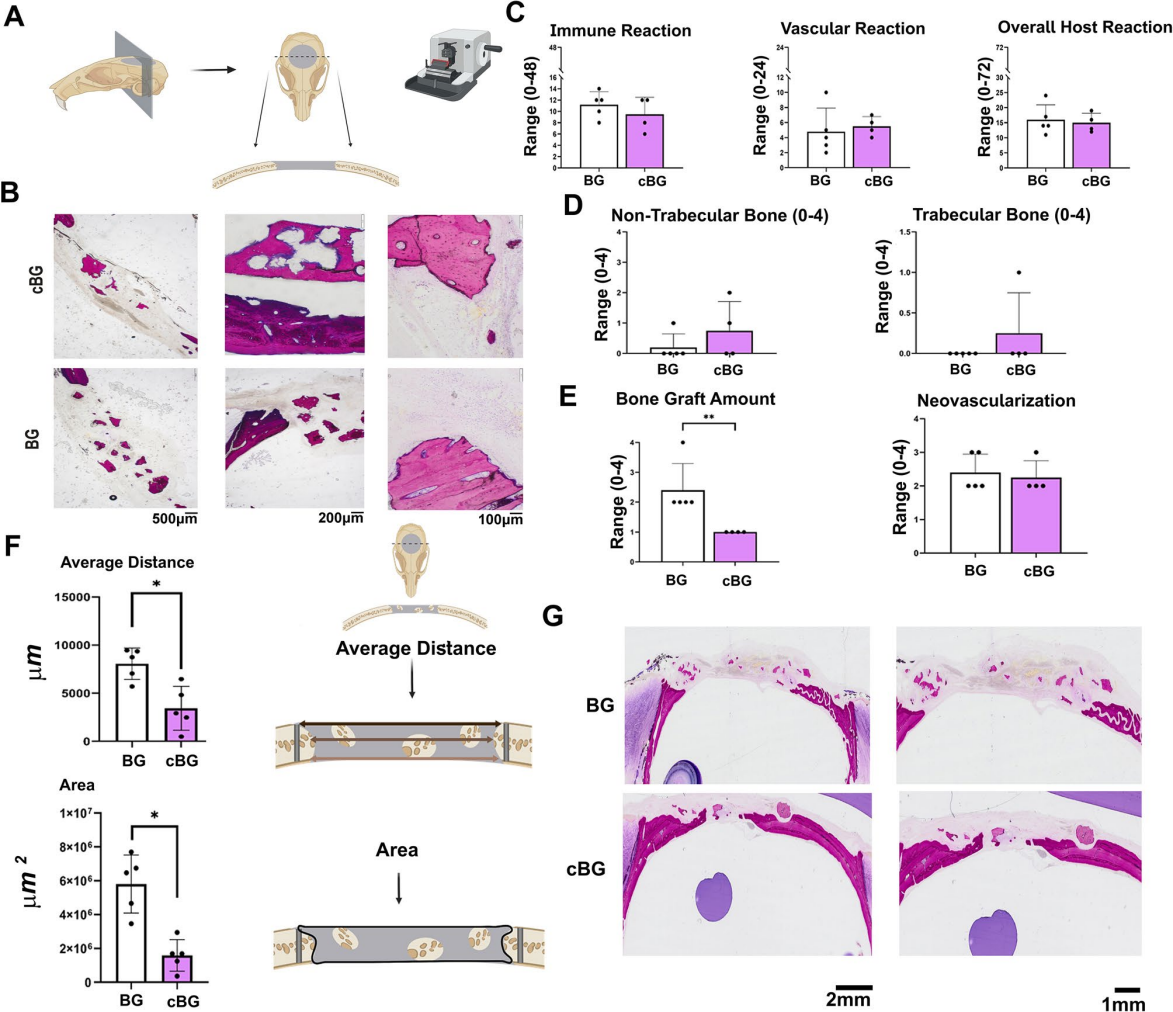
Histological analysis of mid-coronal sections stained with Paragon staining: (**A**) Scheme of the calvarial defect and mid coronal section; (**B**) Representative images of Paragon stained sections, exhibiting the bone graft in the defect site; (**C**) Host reaction scores, including Immune reaction, vascular reaction and overall host reaction (Supplementary Table 3, raw scores). (**D**) Trabecular (right-handed panel) and non-trabecular (left-handed panel) scores of native bone in the defect site; (**E**) Bone graft amount (left-handed panel) and neo-vascularization (right-handed panel) scores; (**F**) Average distance (upper panel) and area (lower panel) between the defect site edges. (**G**) Representative images of distance and area between defect edges. Statistical significance was assessed based on Wilcoxon test (*p* < 0.05 denotes as “*”, n = 5). Panel A,F illustrations were adapted from Carmon et al. 2023^13^.

The histological sections stained with Paragon, were evaluated for mid-coronal section (Scheme, Fig. 5A). The defect area stained with Paragon and the magnified bone graft particles are exhibited in Fig. 5B.

Comparative group evaluation of the average host reaction score, revealed similar host reaction scores for both treatments, related to inflammatory or vascular response (Fig. 5C; average host reaction score of 16.0 for BG and average host reaction score of 15.0 for cBG). While some cases of cBG exhibited higher histopathological scores for non-trabecular and trabecular bone formation, the differences between the groups was insignificant (Fig. 5D). Interestingly, neovascularization appeared similar between the groups (Right graph, Fig. 5E), yet cBG exhibited lesser bone graft amount (Left graph, Fig. 5E).

We next analyzed the Paragon stained histological sections to assess the average distance and area between the edges of the calvarial defect (Scheme in Fig. 5F). The data show that the average distance and area was significantly lower in the cBG group vs BG group (Fig. 5F; representative images in Fig. 5G), supporting that the bone formation was expedited at the edges of the defect, as seen in the mineral apposition distance.

**Figure 6:**
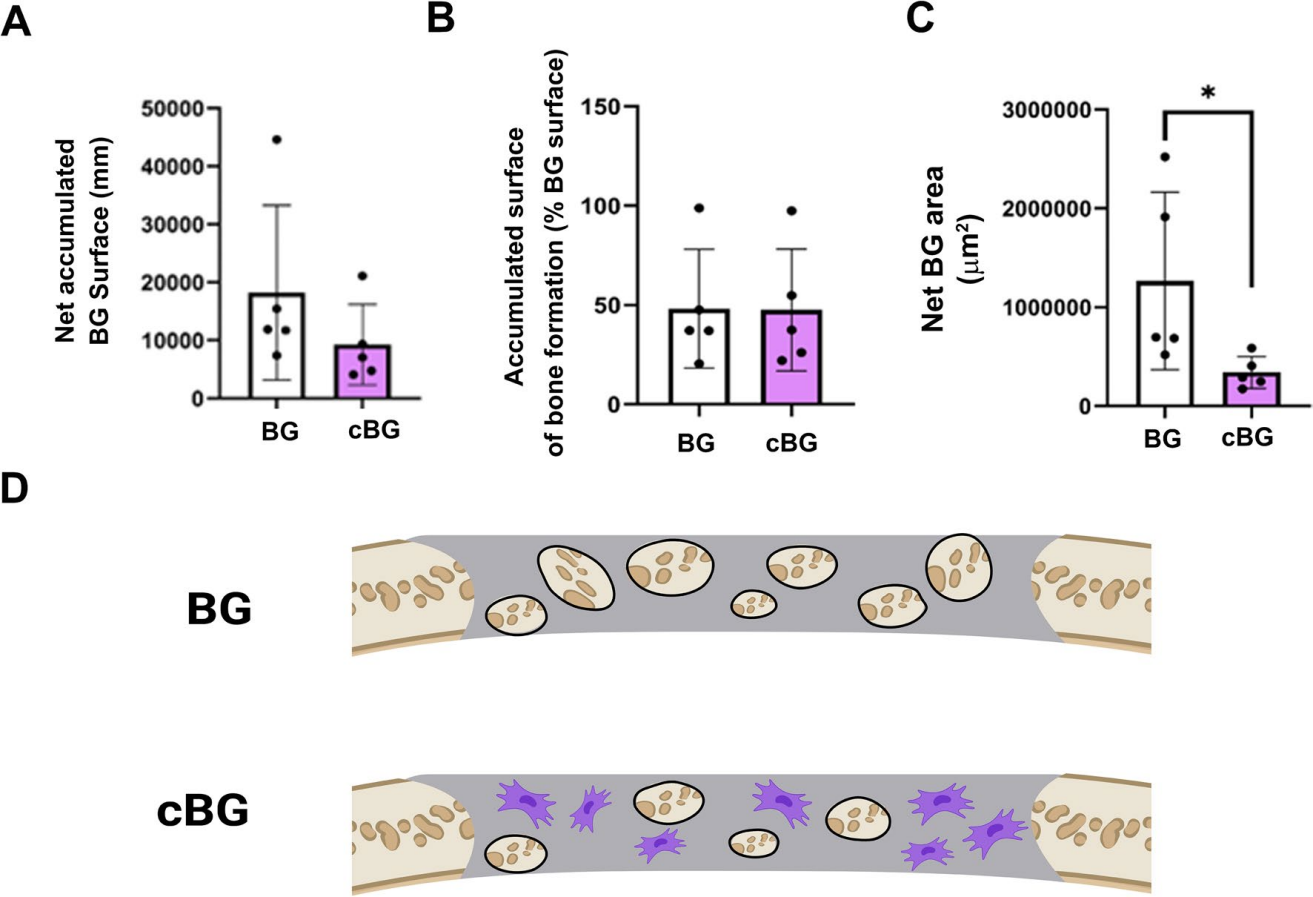
Reduced Bone graft within cBG defect site. (**A**) Paragon-stained slides manually traced the surface around the bone grafts (BG, black lines), excluding the surface surrounding the voids within each bone graft particle, to generate “Net accumulated BG surface (mm)”. (**B**) Denotes the percent of active bone formation surfaces (i.e. displaying either Alizarin complexon or Calcein staining), vs total bone graft surface (%). (**C**) Denotes accumulated net area of bone graft within the defect (µm^2^). (**D**) Exhibits a scheme to show lesser bone graft in the cBG treatment, wherein the purple cells are shown occupying the implant area (grey). Statistical significance was assessed based on Wilcoxon test (*p* < 0.05 denotes as “*”, n = 5). Panel D illustration was adapted from Carmon et al. 2023^13^.

Closer analysis of the bone graft amount within the defect site (Illustration in Fig. 6D), revealed that the net surface of bone graft does not vary between the treatments (Fig. 6A). Moreover, we did not detect differences in the active bone formation (i.e. fluorescently stained red and/or green) surface (Fig. 6B), indicating that the bone graft particles exhibit similar bone formation rates between BG and cBG. However, the accumulated area of bone graft was reduced significantly in the cBG (Fig. 6C). The significant loss of bone graft apparent in cBG group, may indicate that lesser bone graft enters the collagen mesh when chondrocytes are co-applied, as illustrated in Fig. 6D, as they occupy volume within the scaffold pores.

## Discussion

The treatment of calvarial flat bone fractures has been challenging, especially when there is a critical distance between the edges of the fractured bone^2, 21^. Several techniques are available in clinical use to treat fractures or reconstruction of flat bones, such as autologous bone grafting^3^, or 3D-custom printed titanium implants filled with bone substitutes of allografts or xenograft (i.e., DFBA)^9, 10, 22^. Such procedures often fail, due to significant donor site morbidities or complications caused by the titanium implant^5^. To overcome these obstacles, the use of chondrocytes has been reported, as an alternative approach in recent years, especially given the nature of cartilage tissue, which is devoid of nerves and vasculature^11, 12^. As previously reported, chondrocytes may expedite bone repair and calvarial critical size defect^13^, when seeded on top of collagen scaffolds. As another step forward, this study attempts to decipher if this effect is maintained in DFBA-containing collagen implants using a critical size defect model in rats. The use of DFBA is often employed to generate bone due to its capacity to provide adequate mechanical support during the regeneration of bone within a fracture. However, DFBA may contain residual factors^17^, which may affect the capacity of chondrocytes to mineralize^13^, hence this study aimed to assess if the addition of chondrocytes to such constructs may be beneficial in generating osseous matrix.

Initially, we employed a fast extraction protocol of chondrocytes to determine the possibility of such a procedure in clinical practice. Like the reports of Vedicherla and colleagues from 2017^20^, we showed that chondrocytes extraction could be obtained in 30–60 min without significant effects on the viability of the cells. Next, we assessed the healing of calvarial defect, a procedure based on Spicer et al.^19^, when chondrocytes are seeded on collagen scaffold and added to bone graft DFBA implant. Live CT scans throughout the experimental duration showed increased bone volume and strong significant correlation between calcified content and time in cBG. Endpoint microCT evaluation of bone volume confirmed the observations of live CT, although systemic bone turnover markers (i.e., P1NP and CTX-1) which we anticipated to be elevated in the cBG group^23, 24^, didn’t show differences, as compared to BG implanted rats. The histological data confirmed that the majority of bone formation was carried out at the defect edges of the cBG constructs. Indeed, chondrocytes have been reported to exhibit enhanced migration to defect site^25^, inducing repair of osteochondral defects in collagen type I matrices, which may be the case here.

A major limitation of assessing bone formation and micro-structures in CT-based imaging of DFBA containing implants, is the detection of such substances as radiopaque, with similar ossified properties, to that of the regenerated osseous tissue or native bone^26^. Therefore, the histopathology was essential to evaluate the reaction to the graft and profile of bone formation. While bone was formed in the defect edges, the cBG graft appeared to exhibit lesser bone graft area within the defect site, at the experimental end, which was also confirmed upon manual quantification of the graft area within the defect site. One possible explanation is that chondrocyte occupation within the scaffold pores, may eventually reduce the amount of BG within the collagen mesh. While we couldn’t histologically detect osteoclasts or confirm enhanced bone resorption within the defect site, it may be possible that the incorportated chondrocytes stimulated bone resorption. For example, chondromodulin secreted from chondrocytes may stimulate enhanced bone resorption, as its murine knock out presented high bone mass levels^27^. While the exact mechanism by which chondrocytes may contribute to bone formation is unknown from this study, there are several possibilities wherein chondrocytes may transdifferentiate into ostoblasts, or undergo an endochondral-like phase whereby osteoblastic cells/progenitors may be recruited or activated in the presence of chondrocytes. Future work will attempt to label the seeded chondrocytes to determine their viability and fate within the implant, and to determine if they secrete mediators that may stimulate bone related response. Further work should also explore the use of allogenic sources of chondrocytes, some of which may derive from mesenchymal stem cells, embryonic or induced pluripotent stem cells, which undergo controlled chondrogenesis protocols^28, 29^, to modulate an endochondral embryonic state, likely to mineralize and repair such fractures. 

Cumulatively, the data supports that chondrocytes are capable of accelerating bone healing and bone volume increase in calvarial defects, when integrated with bone grafts and therefore serve as a feasible clinical approach for treating such conditions. As mentioned, this study was unable to underpin the cellular mechanism involved in the generation of osseus matrix, albeit that the imaging and histological findings support such matrix is generated upon introducing chondrocytes to the implant. Further work will be required to understand the local cellular mechanisms supporting this process, including osteoclast-mediated resorption, chondrocyte trans-differentiation or stem cell migration. In sum, our results provide support that addition of chondrocytes to DFBA-collagen scaffolds, expedite bone formation and could be employed for the purpose of regenerating osseous matrix in a flat critical bone defect, with minimal donor-site morbidity.

## Conclusions

This study displays that adding chondrocytes to a DFBA/collagen scaffold encourages the generation of mineralized tissue in a critical defect site, through enhanced bone formation at the defect edges. As such, this data provides a foundation for the incorporation of autologous chondrocytes, or stem-cell directed chondrocytes, for the repair of bone defects, requiring surgical intervention.

### Supplementary Information

Supplementary Table 1.

Supplementary Table 2.

Supplementary Table 3.

## Data Availability

The data will be available at the corresponding author website or upon reasonable request.
