# Supplementary material for: Chondrocytes supplemented to bone graft-containing scaffolds expedite cranial defect repair
Source: Sci Rep. 2023 Nov 6;13:19192. doi: 10.1038/s41598-023-46604-z (PMC10628268; doi:10.1038/s41598-023-46604-z)
Supplement: Supplementary file 1 — Supplementary Table 1. [file 41598_2023_46604_MOESM1_ESM.docx]

**Supplemental Table 1: Host Reaction Scoring System for implantation sites adapted to ISO 10993-6:2016(E).**

| **Cell type/response** | **Score** | | | | |
| --- | --- | --- | --- | --- | --- |
|  | **0** | **1** | **2** | **3** | **4** |
| Polymorphonuclear cells | 0 | Rare,1-5/HPF | 5-10/HPF | Heavy infiltrate | Packed |
| Lymphocytes | 0 | Rare,1-5/HPF | 5-10/HPF | Heavy infiltrate | Packed |
| Plasma cells | 0 | Rare,1-5/HPF | 5-10/HPF | Heavy infiltrate | Packed |
| Macrophages | 0 | Rare,1-5/HPF | 5-10/HPF | Heavy infiltrate | Packed |
| Giant cells | 0 | Rare,1-2/HPF | 3-5/HPF | Heavy infiltrate | Sheets |
| Necrosis | 0 | Minimal | Mild | Moderate | Severe |
| **Calculation** | **Subtotal (x2)** | | | | |
| Neovascularization | 0 | Minimal capillary proliferation, focal, 1-3 buds | Groups of 4-7 capillaries with supporting fibroblastic structures | Broad band of capillaries with supporting structures | Extensive band if capillaries with supporting fibroblastic structures |
| Edema | 0 | Minimal | Mild | Moderate | Severe |
| Hemorrhage | 0 | Minimal focal | Mild, focal to multifocal | Moderate, focally extensive | Severe, focally extensive |
| Hemosiderin | 0 | Minimal | Mild | Moderate | Severe |
| **Calculation** | **Subtotal** | | | | |
|  | **Total** | | | | |
|  | **Group Total** | | | | |
|  | **Arithmetic Mean** | | | | |

*HPF= High Power Field

Defined by the ISO 10993-6:2016(E), a score difference between 0.0 to 2.9 is considered no or minimal host reaction, 3.0 to 8.9 slight host reaction, 9.0 to 15.0 moderate host reaction
