# Supplementary material for: Chondrocytes supplemented to bone graft-containing scaffolds expedite cranial defect repair
Source: Sci Rep. 2023 Nov 6;13:19192. doi: 10.1038/s41598-023-46604-z (PMC10628268; doi:10.1038/s41598-023-46604-z)
Supplement: Supplementary file 2 — Supplementary Table 2. [file 41598_2023_46604_MOESM2_ESM.docx]

**Supplemental Table 2: Additional parameters evaluated by histopathology.**

| **Cell type/response** | **Score** | | | | |
| --- | --- | --- | --- | --- | --- |
|  | **0** | **1** | **2** | **3** | **4** |
| Collagen Formation / Deposition | 0 | Collagen formation occupying up to 10% of the defect | Collagen formation occupying from 11 to 20% of the defect | Collagen formation occupying from 20 to 30% of the defect | Collagen formation occupying more than 35% of the defect |
| Fibrosis | 0 | Minimal narrow band along the defect | Mild thick band along the defect | Moderate thick band along the defect | Extensive marked thick band along the defect |
| Non-Trabecular Bone formation (healing) | None to minimal non-trabecular bone around the defect edges | Minimal to slight non-trabecular bone around the defect edges (< 10% of defect length, approximated). Non or minimal focal isolated bone formation at the defect | Minimal to slight non-trabecular bone around the defect edges (10- 33% of defect length, approximated). Minimal to slight focal isolated bone formation at the defect | Minimal to slight non-trabecular bone around the defect edges (>33% of defect length, approximated). Slight to moderate focal to multifocal isolated bone formation at the defect | Severe non-trabecular bone formation and complete defect healing. |
| Trabecular Bone Formation including cavity with bone marrow | Absence | Minimal focal presence of trabecular bone at the defect | Mild focal to multifocal presence of trabecular bone at the defect | Moderate multifocal presence of trabecular bone at the defect | Severe multifocal presence of trabecular bone at the defect |
| Amount of bone graft occupying the defect | 0 | Up to 10% of the defect | From 11 to 20% of the defect | From 20 to 30% of the defect | More than 30% of the defect |
