# Supplementary material for: Chondrocytes supplemented to bone graft-containing scaffolds expedite cranial defect repair
Source: Sci Rep. 2023 Nov 6;13:19192. doi: 10.1038/s41598-023-46604-z (PMC10628268; doi:10.1038/s41598-023-46604-z)
Supplement: Supplementary file 3 — Supplementary Table 3. [file 41598_2023_46604_MOESM3_ESM.docx]

**Supplemental Table 3: Individual Sample Data.**

| **Treatment** | **Sample ID** | **Host Reaction** | | | | | | | | | | | | | | **Additional Parameters** | | | | | |
| --- | --- | --- | --- | --- | --- | --- | --- | --- | --- | --- | --- | --- | --- | --- | --- | --- | --- | --- | --- | --- | --- |
|  |  | Polymorphonuclear cells | Lymphocytes | Plasma cells | Macrophages | Giant cells | Necrosis | **Subtotal (x2)** | Hemorrhage | Neovascularization | Hemosiderin | Edema | **Subtotal** | **Total Host Reaction** | Non-Trabecular Bone (healing) | | Trabecular Bone Formation | Fibrosis | Collagen | Bone Graft Amount |  |
| BG | 8L | 0 | 3 | 0 | 3 | 0 | 0 | **12** | 0 | 2 | 0 | 0 | **2** | **14** | 0 | | 0 | n.a* | n.a* | 2 |  |
|  | 8R | 0 | 1 | 0 | 3 | 0 | 0 | **8** | 0 | 3 | 0 | 0 | **3** | **11** | 1 | | 0 | n.a* | n.a* | 2 |  |
|  | 9L | 0 | 2 | 0 | 3 | 0 | 0 | **10** | 0 | 2 | 2 | 0 | **4** | **14** | 0 | | 0 | n.a* | n.a* | 4 |  |
|  | 27L | 1 | 3 | 0 | 2 | 0 | 0 | **12** | 2 | 3 | 0 | 0 | **5** | **17** | 0 | | 0 | n.a* | n.a* | 2 |  |
|  | 11R | 1 | 3 | 0 | 3 | 0 | 0 | **14** | 4 | 2 | 4 | 0 | **10** | **24** | 0 | | 0 | n.a* | n.a* | 2 |  |
| cBG | 25L | 0 | 2 | 0 | 2 | 0 | 0 | **8** | 2 | 2 | 1 | 0 | **5** | **13** | 2 | | 1 | n.a* | n.a* | 1 |  |
|  | 25R | 1 | 2 | 0 | 3 | 0 | 0 | **12** | 3 | 2 | 2 | 0 | **7** | **19** | 0 | | 0 | n.a* | n.a* | 1 |  |
|  | 26R | 1 | 3 | 0 | 2 | 0 | 0 | **12** | 1 | 2 | 1 | 0 | **4** | **16** | 0 | | 0 | n.a* | n.a* | 1 |  |
|  | 26L | 0 | 2 | 0 | 1 | 0 | 0 | **6** | 2 | 3 | 1 | 0 | **6** | **12** | 1 | | 0 | n.a* | n.a* | 1 |  |
|  | 27R | n.a** | n.a** | n.a** | n.a** | n.a** | n.a** | **n.a** | n.a** | n.a** | n.a** | n.a** | **n.a** | **n.a** | 1 | | 0 | n.a* | n.a* | n.a |  |

n.a.: not applicable

n.a.*: Collagen formation / deposition cannot be separated from fibrosis as of the used collagen tape.

n.a.**: Cellularity not visible.

Additional Remarks:

25R - Focal adherence of fibrous tissue to the brain.

27R - Section slightly to the side. Scoring was adapted to reflect less central sectioning level of the defect, as the defect diameter/distance will be less and the healing is more prominent on the sides.
